# Prognostic Outcomes and Risk Factors for Patients with Renal Cell Carcinoma and Venous Tumor Thrombus after Radical Nephrectomy and Thrombectomy: The Prognostic Significance of Venous Tumor Thrombus Level

**DOI:** 10.1155/2015/163423

**Published:** 2015-09-03

**Authors:** Qi Tang, Yi Song, Xuesong Li, Maxwell Meng, Qian Zhang, Jin Wang, Zhisong He, Liqun Zhou

**Affiliations:** ^1^Department of Urology, Peking University First Hospital, Institute of Urology, Peking University, National Urological Cancer Center, Beijing 100034, China; ^2^Department of Urology, University of California, San Francisco, CA 94143-0738, USA; ^3^Department of Cardiac Surgery, Peking University First Hospital, Beijing 100034, China

## Abstract

*Introduction*. To evaluate the prognostic outcomes and risk factors for renal cell carcinoma (RCC) patients with venous tumor thrombus in China. *Materials and Methods*. We reviewed the clinical information of 169 patients who underwent radical nephrectomy and thrombectomy. Overall and cancer-specific survival rates were analyzed. Univariate and multivariate analyses were used to investigate the potential prognostic factors. *Results*. The median survival time was 63 months. The five-year overall survival and cancer-specific survival rate were 53.6% and 54.4% for all patients. For all patients, significant survival difference was only observed between early (below hepatic vein) and advanced (above hepatic vein) tumor thrombus. However, significant differences existed between both RV/IVC and early/advanced tumor thrombus groups in N0M0 patients. Multivariate analysis demonstrated that higher tumor thrombus level (*p* = 0.016, RR = 1.58), N (*p* = 0.013, RR = 2.60), and M (*p* < 0.001, RR = 4.14) stages and adrenal gland invasion (*p* = 0.001, RR = 4.91) were the most significant negative prognostic predictors. *Conclusions*. In this study, we reported most cases of RCC patients with venous extension in China. We proved that patients with RCC and venous tumor thrombus may have relative promising long-term survival rate, especially those with early tumor thrombus.

## 1. Introduction

Renal cell carcinoma (RCC) represents approximately 5% of new cancer cases in the United States and is the third most common cancer of the urinary system [1]. According to the 2012 Chinese Cancer Registry Annual Report, RCC accounts for 2.2% of cancer cases in China [2]. Tumor extension to the venous system, including the renal vein (RV) and the inferior vena cava (IVC), is a feature of locally advanced RCC and occurs in approximately 4–10% of cases [3].

The management of RCC patients with venous involvement has changed in recent decades. With advanced surgical techniques, particularly the development of cardiopulmonary bypass, more surgeons are able to surgically remove the kidney and attached tumor thrombus as the primary management approach [4–7]. Recently, even laparoscopic techniques have been introduced into surgical strategies for these patients because of their advantages in kidney mobilization and blood loss control [8, 9]. It is well documented that patients with RCC and venous tumor thrombus may significantly benefit from radical surgical resection, even those with distant metastasis [5]. Although there are data reporting the long-term outcomes of these patients worldwide, reliable statistics based on a large number of patients in China remain lacking.

Some studies have researched the prognostic significance of many factors in RCC patients with venous extension, such as tumor size, tumor thrombus level, histological subtype, Fuhrman grade, nodal status, and distant metastasis [10–13]. However, the significance of venous involvement and tumor thrombus level still remain controversial. RCCs with venous tumor thrombus are aggressive tumors belonging to stage T3 and are associated with poor prognosis [14]. Some investigators reported that patients with higher tumor thrombus levels may have a poorer long-term outcome [7, 12]. However, other studies demonstrated no significant worsened survival between different thrombus levels [14, 15]. In addition to tumor size, thrombus level, and pathological features, the negative effect of tumor perinephric fat invasion on the prognosis of RCC patients with venous involvement has also been recognized recently [11, 16, 17].

The objectives of the present study were to report our experience in the surgical management of RCC patients with venous tumor thrombus, to assess the overall survival and cancer-specific survival rate of Chinese patients and to evaluate the significance of potential prognostic factors, especially the venous tumor thrombus level. To our knowledge, this is the largest report of patients with RCC and venous tumor extension in China.

## 2. Materials and Methods

### 2.1. Patient Selection

We retrospectively reviewed the records of all RCC patients treated in our hospital, from August 2000 to December 2012. Patients with RCC and venous tumor thrombus who were treated with radical nephrectomy and tumor thrombectomy were included. Patients with incomplete medical records or palliative surgical resection were excluded.

### 2.2. Clinical and Pathology Information

All patients had preoperative routine blood examinations, chest X-rays, electrocardiograms, and abdominal computerized tomography or magnetic resonance imaging evaluations. The venous tumor extension was indicated by preoperative radiological examination and confirmed during surgery procedure. To define the level of venous tumor thrombus extension, we followed the Neves classification system [18]. The first level of tumor thrombus includes those that are restricted to renal vein. Level I IVC thrombus refers to those that extend into inferior vena cava but below the hepatic veins. Level II IVC thrombus represents those that extend above the hepatic veins but below the diaphragm, and level III IVC thrombus is defined as a tumor thrombus that extends above the diaphragm or into the right atrium. Given the diversity of surgical strategies, we classified patients into early and advanced venous tumor thrombus, using the hepatic vein as the cut-off line. The venous tumor thrombus levels for all patients were decided comprehensively by preoperative radiologic information, including computerized tomography, magnetic resonance imaging, transesophageal echocardiography, and exploration during surgery. Preoperative distant metastasis status was routinely confirmed by chest X-rays, chest CT, and abdominal ultrasound. Cranial MRI or bone scans may also be performed if the patients had relevant symptoms. Postoperative immunotherapy or targeted molecular therapies were suggested if distant metastasis existed before surgery.

A multidisciplinary consultation, including specialists from urology, general surgery, cardiac surgery, anesthesiology, and radiology departments, will give a comprehensive assessment of the patient before surgery. For patients who cannot tolerate the surgery, palliative therapies may be suggested rather than radical resection.

To evaluate the potential prognostic factors for patients with RCC and venous tumor extension, clinical information such as age, gender, tumor size, first symptoms, operation time, blood loss, pathology subtype, Fuhrman grade, perinephric fat invasion, and 2009 TNM stage was also reviewed.

### 2.3. Surgery and Complications

A total of 169 patients completed radical nephrectomy and thrombectomy. Urologists, cardiac surgeons, and anesthetists jointly decided whether extracorporeal circulation was required based on the upper limit of tumor thrombus and the estimation of collateral circulation. Extracorporeal circulation was mainly used in patients with thrombus extension beyond the hepatic vein. Cardiopulmonary bypass with deep hypothermia circulatory arrest was seldom used in our institute because of high complication rate. Extended lymph node dissections were not routinely performed, but we performed lymphadenectomy for N staging if enlarged regional lymph nodes were observed in preoperative imaging or during the operation procedure. Gentle manipulations were always emphasized in our operations, especially around the renal vein and inferior vena cava. They would help decrease the risk of pulmonary embolism caused by tumor thrombus detachment. Recently, we introduced a laparoscopic technique for the surgical management of patients with venous involvement. We demonstrated that the combination of laparoscopic nephrectomy and open thrombectomy could decrease the operation time and blood loss.

The perioperative complications were reviewed. Complications which needed no intervention or only pharmacological treatment were defined as minor complications, while major complications referred to those that needed invasive intervention, life-threatening complications, or even perioperative death.

### 2.4. Monitoring and Follow-Up

Patients were required to complete routine laboratory tests and imaging assessment (including chest X-rays, urinary system ultrasound, and abdominal CT/MRI) every 3 months in the first two years, every 6 months in the subsequent year, and once a year in the following years. Appropriate treatments were provided in cases of local recurrence or distant metastasis. Follow-up information was obtained from phone interviews and outpatient records. The last follow-up was completed in December 2013. During follow-up, the cause of patient's death was confirmed by the death certificate offered by the hospital.

### 2.5. Statistical Analysis

Continuous parametric and nonparametric variables were reported differently as the mean value ± SD and median values. Continuous variables were analyzed using Student's *t*-test (normally distributed data) and Mann-Whitney *U* test (nonnormally distributed data). Categorical variables were compared using the Pearson *χ*2 test. The survival time was calculated from the date of operation to death or the date of last follow-up (when the patient was confirmed to be alive). The Kaplan-Meier method was used to analyze the survival curve for different tumor thrombus levels, and differences between groups were tested using the log-rank test. We used the Cox proportional hazard model to evaluate multiple predictors of outcome and to eliminate confounding factors. A two-sided *p* value <0.05 was considered to be statistically significant. All data were collected and analyzed by SPSS 20.0 software (IBM Corp, Armonk, NY, USA).

## 3. Results

### 3.1. Clinical and Pathological Characteristics

A total of 169 RCC patients with venous tumor thrombus and radical nephrectomy with thrombectomy were included in our study. The patients consisted of 119 men (70.4%) and 50 women (29.6%), with a median age of 58 years (range 15 to 80). The patients' clinical characteristics were summarized (Table 1). There were 93 (55.0%) patients with RV tumor thrombus and 76 (45.0%) with IVC tumor thrombus including 49 with level I, 13 with level II, and 6 with level III IVC tumor thrombus. The pathological features of all patients were listed in Table 2.

### 3.2. Surgical Strategy

The patients' surgical characteristics were also summarized in Table 1. There were 24 patients who required a cardiopulmonary bypass during surgery, including 1 with IVC level I, 17 with IVC level II, and 6 with IVC level III tumor thrombus. The laparoscopic technique was used for 19 patients all of whom had tumor thrombus below the hepatic vein, including 14 patients with pure laparoscopic surgery and 5 patients with combined laparoscopic and open surgery.

### 3.3. Perioperative Complications

The complication rate was 37.3% (63/169), including 47 minor complications and 16 major complications. The most common postoperative minor complication was transient renal insufficiency which needed no dialysis. One patient suffered from ileus and did not recover after conservative therapies and underwent a reoperation on the twenty-fifth day after the first surgery. Three patients had solitary kidney before surgery and required routine dialysis after the radical resection. Four patients experienced postoperative pulmonary embolism and one of them died because of heart failure and severe pulmonary infection.

### 3.4. Survival and Prognosis Factors

The median follow-up time was 45 months (2–114 months). The survival information of 143 patients was available, with a follow-up rate of 84.6% (143/169). At the last follow-up, 52 patients deceased, including 49 cancer-related deaths and 3 deaths unrelated to RCC (cerebral infarction, primary hepatic carcinoma, and interstitial pneumonia). Eleven patients were alive with tumor progression, while 80 patients were alive and disease-free at the last follow-up. The estimated median overall survival (OS) was 63.0 months for all patients. The 3-year and 5-year OS were 73.4% and 53.6%, while the 3-year and 5-year cancer-specific survival (CSS) were 74.4% and 54.4%. There were 96 patients (67.1%, 96/143) who had no evidence of initial nodal and distant metastasis (N0M0). For the N0M0 subgroup patients, the 5-year OS and CSS were 71.8% and 73.2%.

**Figure 1:**
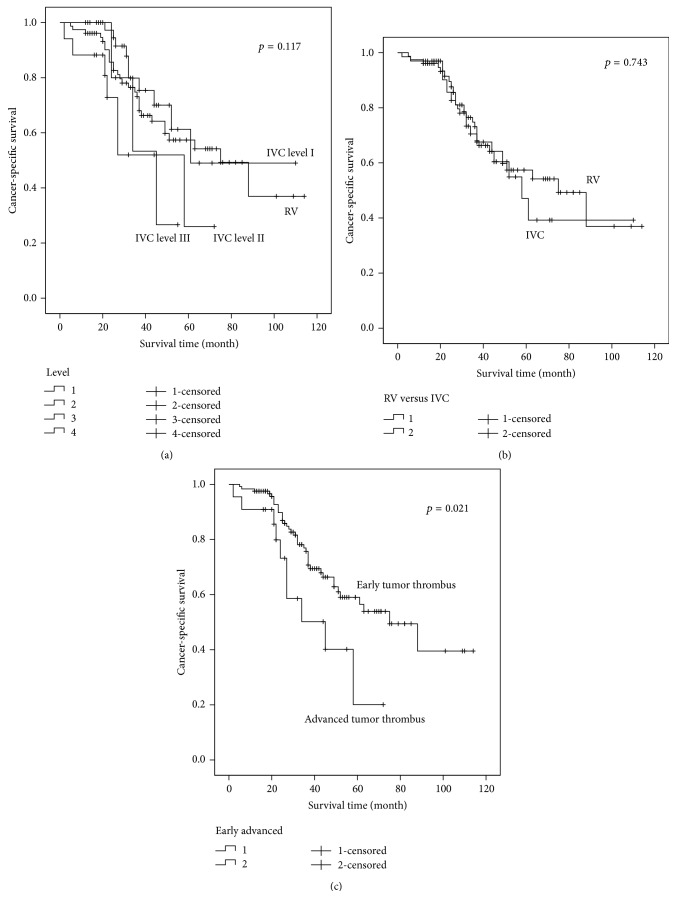
Prognosis comparison between patients with different tumor thrombus levels. (a) No significant difference between four tumor thrombus levels (*p* = 0.117). (b) No significant difference between RV and IVC tumor thrombus (*p* = 0.743). (c) Significantly better prognosis for the patients with early tumor thrombus, compared to the advanced patients (*p* = 0.021).

**Figure 2:**
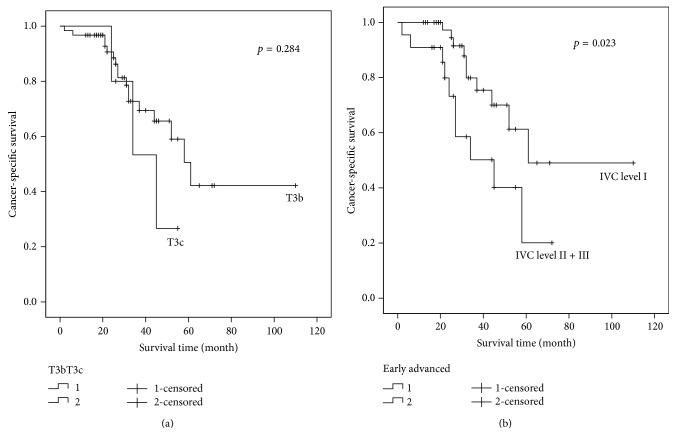
Prognosis comparison between T3 subgroups. (a) T3b and T3c patients had no significantly different long-term survival (*p* = 0.284). (b) Patients with IVC level II or level III tumor thrombus had significantly worse prognosis than those with IVC level I tumor thrombus (*p* = 0.023).

The median CSS of different tumor thrombus levels was 75 months (RV), 61 months (IVC level I), 58 months (IVC level II), and 45 months (IVC level III). For all patients, there was no significant difference of CSS between neither the four tumor thrombus levels (*p* = 0.117) nor the RV and IVC tumor thrombus (*p* = 0.743), while statistically significant CSS difference existed between the early and the advanced tumor thrombus groups (*p* = 0.021) (Figure 1). However, when considering the N0M0 subgroup, whether between the four different tumor thrombus levels, between RV and IVC tumor thrombus, or between the early and the advanced tumor thrombus, all CSS showed significant differences (*p* = 0.011, 0.036, and 0.004). According to the 2009 TNM classification, T3b and T3c patients were divided by the diaphragm. We compared the prognosis of T3b (IVC level I and level II) and T3c (IVC level III) patients, and no difference was found (*p* = 0.284) (Figure 2(a)). However, when taking the hepatic vein as the cut-off line, significant difference of prognosis was found between IVC level I and IVC level II + III patients (*p* = 0.023) (Figure 2(b)).

Univariate and multivariate analyses results were demonstrated (Tables 3 and 4). In the univariate analysis, higher T (*p* = 0.001), N (*p* < 0.001), and M (*p* < 0.001) stages, Fuhrman grade (*p* = 0.045), and adrenal gland invasion (*p* < 0.001) were negative prognostic predictors for all patients. While in the multivariate analysis, independent prognostic risk factors were higher tumor thrombus level (*p* = 0.015, HR = 1.58), N (*p* = 0.013, HR = 2.60) and M (*p* < 0.001, HR = 4.14) stages, and adrenal gland invasion (*p* < 0.001, HR = 4.91).

## 4. Discussion

Various publications have reported that the 5-year survival rate for RCC patients with venous tumor thrombus ranges from 18% to 57% [4–7, 10, 19–21]. However, this does not include data for Chinese RCC patients. To our knowledge, this study is the largest survey of Chinese RCC patients with venous involvement to report long-term outcomes after surgery. By summarizing the clinical and histological features, we analyzed the potential prognostic predictors of these patients and focused on the survival outcome of patients with different levels of venous tumor extension.

The concomitant involvement of the renal vein (RV) and the inferior vena cava (IVC) at RCC diagnosis is relatively rare. In previous years, because of the high surgical complication rate and mortality, patients were always conservatively treated but had poor outcomes [5]. Surgical resection has become a first-line treatment for these patients in recent decades due to the development of improved surgical techniques and medical facilities. The 3-year and 5-year overall survival rates of all patients in our study are 73.4% and 53.6%, which are comparable to the data from previous reports. For the N0M0 patients subgroup, the survival rates are even higher. Our study confirms the validity for using surgical resection in RCC patients with venous involvement at our institute.

The prognostic predictors for RCC patients with venous extension have been extensively analyzed, especially the level of tumor thrombus, which is still controversial. Some studies have demonstrated a decreased survival rate in patients with tumor thrombus extending into the IVC, when compared with RV involvement alone [7]. However, other researchers have not demonstrated that tumor thrombus level is a negative predictor [14, 19, 22]. In our study, for all patients, significant difference of CSS is only found between the early and advanced tumor thrombus groups (*p* = 0.021), when taking the hepatic vein as a cut-off line. Nevertheless, results of the N0M0 subgroup are different. Regardless of being between four different tumor thrombus levels, RV and IVC tumor thrombus, or early and advanced tumor thrombus, significant differences are found (*p* = 0.011, 0.036, and 0.004). Patients with higher tumor thrombus level tend to have worse prognosis. In the multivariate analysis, higher tumor thrombus level is also proved to be an independent prognostic risk factor whether for all patients or N0M0 patients.

The optimal stratification of venous tumor extension is still controversial. The classification of T3 subgroups has been changing over years. In the latest 2009 TNM system, diaphragm is taken as a division of patients with IVC tumor thrombus. In our study, we do not find significant survival difference between T3b and T3c patients. However, when compared to patients with tumor thrombus above the hepatic vein, significant benefit of prognosis is found in IVC level I patients. While considering the results mentioned above, it seems that the hepatic vein may be a more appropriate cut-off line instead of the diaphragm. So we propose the reclassification of T3 subgroups into T3b with IVC tumor thrombus below hepatic vein and T3c with IVC tumor thrombus above it.

We have found no significant predictive effect related to the status of perinephric fat invasion in all patients or in the N0M0 subgroup, which has been demonstrated in some previous studies [11, 20].

Within the last 3 years, we combined laparoscopic-assisted nephrectomy with open thrombectomy in carefully selected patients. Patients initially undergo laparoscopic transretroperitoneal nephrectomy in a lateral position and are then moved to a supine position for open thrombectomy. This surgical combination with laparoscopy allows for smaller incisions compared with a typical open surgical approach. Additionally, laparoscopy allows for clearer visualization and eases the manipulation of the renal pedicle and the abundant collateral vessels. In our experience, the combination of laparoscopic surgery decreases blood loss and shortens the operation time [23, 24]. The laparoscopic manipulation of lumbar veins is crucial. We should make sure that the circular dissection of IVC is completed and all the lumbar veins are ligated, in order to prevent unnecessary blood loss.

In our study, approximately 25% of patients had distant metastasis before surgery. For these patients, postoperative immunotherapy and targeted molecular therapy were suggested. The postoperative therapy information was also collected during follow-up (data not shown), but some patients were still undergoing clinical trials where the exact therapy strategies were not available. In recent years, the effectiveness of neoadjuvant and postoperative targeted molecular therapy for RCC patients with venous tumor thrombus has been reported [25–27]. With the combination of targeted therapy and surgery, patient may obtain an even better prognosis. However, evidences of high quality are urgently needed in this area.

Our study has some limitations. Because RCCs with venous extension are relatively uncommon, especially the lack of patients with advanced tumor thrombus (IVC level II and III IVC thrombus), we may require a consortium that includes more institutes to accumulate more patient data and attain more representative results. As our study is retrospective, we cannot collect patient information following a uniform approach, which may inevitably lead to some bias. Our study covered a long time period. There may be deviations for several variables, such as the pathological criterion of perinephric fat invasion. Nonetheless, to our knowledge, our study represents the largest group of Chinese RCC patients with venous tumor thrombus and fills a void in this area.

## 5. Conclusions

In our study, we confirmed the effectiveness of surgical resection for RCC patients with venous tumor thrombus, with a promising long-term survival rate, which was comparable to previous studies. Higher tumor thrombus level was proved to be an important prognostic risk factor for these patients. Furthermore, the hepatic vein seems to be a more appropriate cut-off line for T3b and T3c tumor thrombus.

## Figures and Tables

**Table 1:** Clinical and surgical characteristics of patients.

	RV	IVC level I	IVC level II	IVC level III	*p*
Patients (*n*)	93	49	21	6	
Male (%)	75.3 (70/93)	59.2 (29/49)	71.4 (15/21)	83.3 (5/6)	0.219
Age (year)	59.5 ± 9.7	54.4 ± 12.1	55.1 ± 10.2	54.7 ± 10.0	0.03
Symptoms					0.491
Yes	59	36	15	3	
No	34	13	6	3	
Tumor position					<0.001
Left	60	11	2	3	
Right	33	38	19	3	
Operation time (hour)					<0.001
Median	3.0	4.0	7.8	8.8	
Range	1.4–6.5	2.0–12.0	4.5–11.5	7.5–13.0	
Blood loss (mL)					<0.001
Median	400	700	3000	2700	
Range	30–6850	100–12800	500–12000	1800–4000	
Blood transfusion (mL)					<0.001
Median	0	350	1550	2700	
Range	0–4250	0–6500	0–7400	1900–10000	

**Table 2:** Pathological features of patients.

	RV	IVC level I	IVC level II	IVC level III	*p*
Tumor size (cm)					0.499
Average	8.9 ± 2.8	8.5 ± 2.8	9.1 ± 2.9	9.6 ± 3.2	
Perinephric fat invasion					0.096
Yes	54	21	10	1	
No	39	28	11	5	
Adrenal gland invasion					0.161
Yes	8	0	2	0	
No	85	49	19	6	
Number of N classifications					0.849
0	82	42	17	5	
1	11	7	4	1	
Number of M classifications					0.132
0	69	42	16	6	
1	24	7	5	0	
Histological subtype					0.446
Clear cell	87	41	16	6	
Papillary	4	3	3	0	
Chromophobe	1	2	1	0	
Collecting duct	0	2	1	0	
Unclassified	1	1	0	0	
Sarcomatoid					0.942
Yes	23	12	6	1	
No	70	37	15	5	
Fuhrman grade					0.613
G2	36	15	6	3	
G3	53	31	12	3	
G4	4	3	3	0	

**Table 3:** Univariate analysis of prognostic risk factors.

Covariate	HR (95% CI)	*p*	HR (95% CI)	*p*
	All patients		N0M0 patients	
Gender	0.99 (0.54–1.80)	0.971	1.12 (0.45–2.82)	0.807
Age	1.00 (0.97–1.03)	0.949	0.98 (0.95–1.02)	0.373
Symptoms	1.73 (0.90–3.31)	0.102	1.45 (0.56–3.79)	0.446
Tumor position	0.81 (0.46–1.42)	0.461	0.90 (0.37–2.16)	0.806
Preoperative embolization	1.18 (0.65–2.11)	0.592	1.37 (0.54–3.43)	0.507
Tumor size	1.06 (0.96–1.17)	0.242	1.03 (0.88–1.21)	0.705
Tumor thrombus level	1.26 (0.91–1.75)	0.167	1.91 (1.24–2.95)	0.003
Perinephric fat invasion	1.08 (0.62–1.90)	0.783	0.59 (0.23–1.48)	0.259
Adrenal gland invasion	5.65 (2.59–12.35)	<0.001	3.14 (0.659–14.933)	0.151
T	1.65 (1.24–2.21)	0.001	1.92 (1.25–2.94)	0.003
N	4.94 (2.51–9.74)	<0.001		
M	4.35 (2.45–7.72)	<0.001		
Histological subtype	1.021 (0.87–1.21)	0.803	0.90 (0.67–1.22)	0.509
Fuhrman grade	1.74 (1.01–3.00)	0.045	1.54 (0.65–3.66)	0.327

**Table 4:** Multivariate analysis of prognostic risk factors.

Covariate	HR (95% CI)	*p*	HR (95% CI)	*p*
	All patients		N0M0 patients	
Gender	0.98 (0.49–1.95)	0.954	0.88 (0.28–2.76)	0.830
Age	0.99 (0.96–1.02)	0.391	0.99 (0.95–1.04)	0.714
Symptoms	1.22 (0.57–2.62)	0.611	2.28 (0.74–7.01)	0.152
Tumor size	1.06 (0.95–1.18)	0.280	1.07 (0.90–1.28)	0.459
Tumor thrombus level	1.58 (1.09–2.29)	0.016	2.12 (1.37–3.27)	0.001
Perinephric fat invasion	1.85 (0.97–3.52)	0.060	0.59 (0.21–1.66)	0.321
Adrenal gland invasion	4.91 (1.97–12.22)	0.001	8.04 (1.38–46.98)	0.021
N	2.60 (1.23–5.51)	0.013		
M	4.14 (2.17–7.93)	<0.001		
Histological subtype	0.94 (0.78–1.13)	0.491	0.85 (0.62–1.16)	0.305
Fuhrman grade	1.21 (0.66–2.21)	0.542	2.29 (0.88–5.98)	0.090
